# Effect of *Flammulina velutipes* polysaccharide on mitochondrial apoptosis in lung adenocarcinoma A549 cells

**DOI:** 10.1038/s41598-024-57211-x

**Published:** 2024-07-12

**Authors:** Fei Zhao, Dan-yang Chen, Bo Jing, Yu Jiang, Lan-yue Liu, Hui Song

**Affiliations:** 1 School of Life Science, Jilin Agricultural University, Changchun, 130118 Jilin People’s Republic of China; 2 Institute of Special Animal and Plant Sciences, Chinese Academy of Agricultural Sciences, 4899 Juye Street, Changchun, 130112 Jilin People’s Republic of China; 3 Engineering Research Center of Chinese Ministry of Education for Edible and Medicinal Fungi, Changchun, 130118 People’s Republic of China

**Keywords:** *Flammulina velutipes* polysaccharide, Mitochondrial pathway, Apoptosis, A 549 cell, Biochemistry, Biological techniques

## Abstract

FVP is a polysaccharide extracted from *Flammulina velutipes* with immunomodulatory, anti-tumor, and anti-oxidation activities. In this study, we obtained the crude polysaccharide FVP-C from the water extract of *Flammulina velutipes*, and its main component FVP-S1 was obtained after further purification. Upon structural identification, we found that FVP-C is a neutral polysaccharide, and FVP-S1 was an acidic golden mushroom polysaccharide, consisting of glucuronic acid, xylose, and glucose. Lung adenocarcinoma (A549) was treated with FVP-S1 and FVP-C, respectively, and we found that FVP-S1 and FVP-C inhibited the proliferation and migration ability of tumor cells, as well as changed the morphology of the tumor cells and caused chromosome sheteropythosis, among which FVP-S1 had the best inhibition effect. The results of flow cytometry experiments and mitochondrial membrane potential, RT-qPCR, and Western blot showed that FVP-S1 and FVP-C were able to decrease the mitochondrial membrane potential, increase the expression level of apoptotic proteins Casepase-3 and Casepase-9 proteins, and at the same time, increase the ratio of Bax and Bcl-2, which promoted apoptosis of tumor cells. In conclusion, these data indicated that FVP-S1 and FVP-C were able to induce apoptosis in A549 cells through the mitochondrial pathway, which played an important role in inhibiting tumor cells.

## Introduction

Lung cancer is one of the most common malignant tumors and also contributed to the largest cause of cancer death, with about 1.6 million deaths from lung cancer each year^1^. Its type can be divided into small-cell lung cancer and non-small cell lung cancer, rather than a high incidence of small cell lung cancer, accounting for about 85% of all patients with lung cancer^2^. Traditional treatments for lung cancer include surgery and chemotherapy. However, chemotherapy has serious side effects that can lead to severe adverse reactions^3^. Meanwhile, drug resistance is the most difficult problem in chemotherapy for lung cancer^4^. Therefore, the development of anti-tumor drugs with small side effects and good therapeutic effects will always be one of the research contents of lung cancer treatment.

Polysaccharides are formed by the dehydration and condensation of multiple monosaccharide molecules, a class of saccharides with complex and large molecular structures^5^. Studies have found that natural polysaccharides or polysaccharide complexes isolated from fungi have various biological activities such as anti-tumor and immune regulation^6,7^. Among them, polysaccharides from *Ganoderma applanatum*^8^ and *Grifola frondosa*^9^ have the effects of inhibiting tumor cell proliferation and regulating tumor cell apoptosis, while *Flammulina velutipes*^10^ polysaccharides and *Cordyceps militaris*^11^ polysaccharides play a major role in liver and lung cancer.

Currently, most treatments for tumor cells are aimed at inducing apoptosis using targeted therapy^12^. There are three main ways of apoptosis in tumor cells, including the mitochondrial pathway, the death receptor pathway, and the endoplasmic reticulum pathway, of which the mitochondrial pathway is one of the important pathways of apoptosis^1,13–15^. A previous study showed that *Flammulina velutipes* polysaccharides were able to induce apoptosis in tumor cells through the endoplasmic reticulum pathway. For example, Ding, et al.^16^. found that the *Flammulina velutipes* polysaccharide FVP was able to induce ERS-mediated apoptosis by activating the PLC-IP3 pathway in HepG2 cells^16^. Meanwhile, Chang, et al.^17^. found that immunomodulatory proteins in the *Flammulina velutipes* were able to activate host innate and adaptive immunity to trigger cytotoxic immune responses, showing antitumor activity. immunity and adaptive immunity to trigger cytotoxic immune responses, showing anti-tumor activity^17^. However, there are few studies about crude polysaccharides and polysaccharides in the induction of apoptosis in tumor cells through the mitochondrial pathway. Therefore, this study will investigate the role of polysaccharides from the same source with different degrees of purification on the regulation of apoptosis in A549 cells, which will provide a useful reference for the comparison of the anti-tumor effects of polysaccharides and crude polysaccharides.

## Results

### Isolation, purification and characterization of FVP-S1 and FVP-C

**Figure 1:**
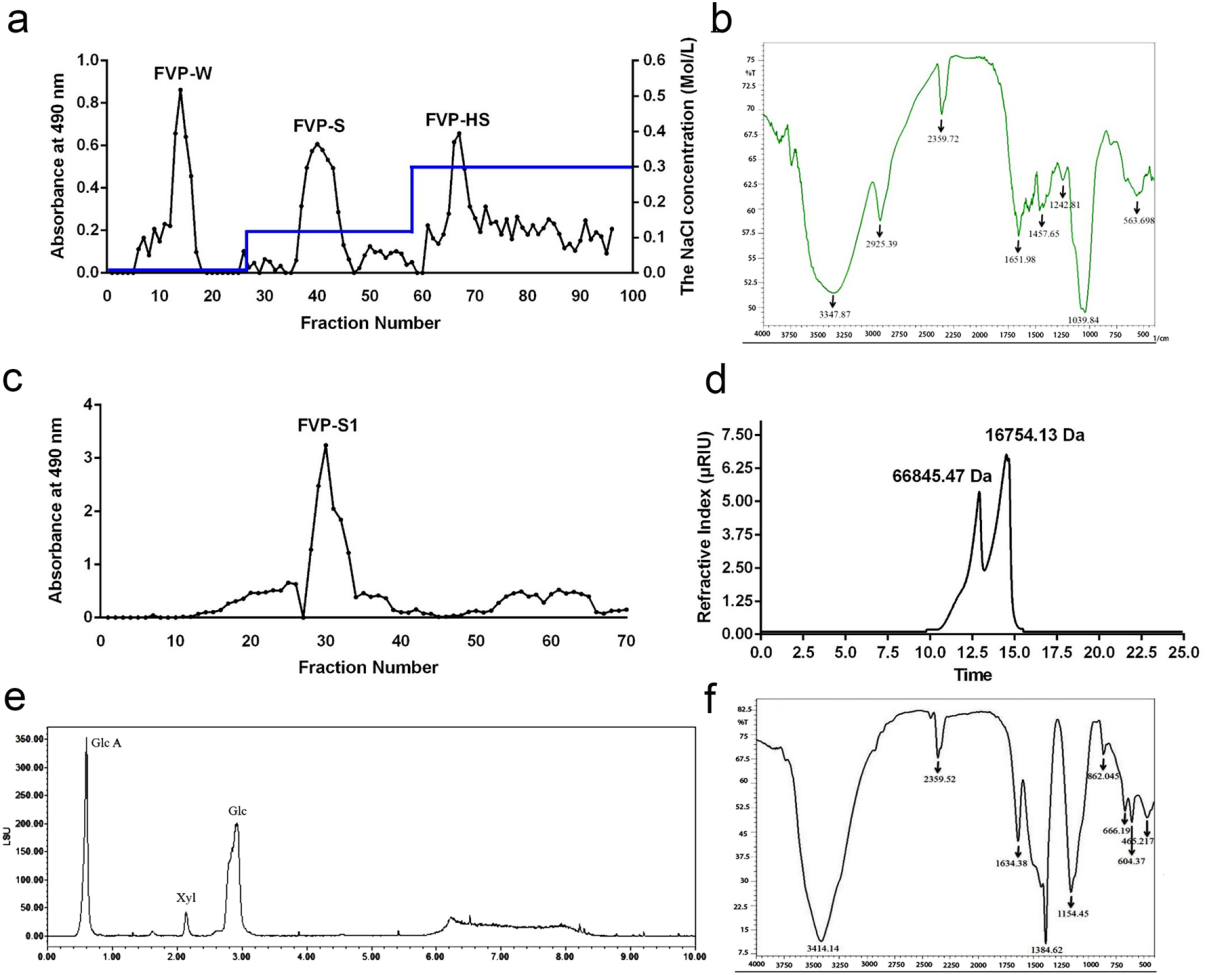
Structural characterization of FVP-C and FVP-S1. (**a**) Resolution of the crude polysaccharide preparation FVP-C (obtained from ethanol extraction) by DEAE-Sepharose Fast Flow column chromatography. (**b**) FTIR spectrum of FVP-C. (**c**) Resolution of FVP-S1 by Sephadex G-100 column chromatography. (**d**) HPGPC file of FVP-S1. (**e**) HPLC profile of hydrolysate of FVP-S1. (**f**) FTIR spectrum of FVP-S1.

We extracted the water-soluble polysaccharide FVP-C from *Flammulina velutipes* (Fig. 1a)*.* Analyzing the chemical composition of FVP-C we found that its total sugar content was found to be 41.57 ± 2.99%, and the reducing sugar content was 0.02 ± 0.01%. The protein content was 0.05 ± 0.01%, the uronic acid content was 0.21 ± 0.01%, and the sulfate group content was 0.98 ± 0.14%, indicating that FVP-C is a neutral polysaccharide (Table. 1). By IR spectral analysis, FVP-C was found to have an O–H bond characteristic vibration at 3347.87 cm^−^1, a C–H bond characteristic vibration at 2925.39 cm^−1^, the C=O bond characteristic vibration with a possible carboxyl group at 1651.98 cm^−1^, and a β-glucopyranose ring in the glucose residue at 1039.84 cm^−1^, at 563.698 cm^−1^ is a characteristic vibration of pyranosidic bonding (Fig. 1b). On further analysis we found that FVP-S1 accounted for the largest proportion of FVP-C, so we further purified to obtain FVP-S1, the HPGPC chromatogram shows that FVP-S1 consists of two polysaccharides with molecular weights of 66,845.47 Da and 16,754.13 Da (Fig. 1c,d). The results of HPLC chromatogram showed that FVP-S1 was mainly composed of glucuronic acid, xylose and glucose. This result indicates that FVP-S1 is an acidic polysaccharide (Fig. 1e). The infrared spectrum results of FVP-S1 are shown in (Fig. 1f), showing a typical strong broadband at 3414.14 cm^−1^ and a weak band at 2359.52 cm^−1^, which are OH bond and NH bond stretching vibration. The bending vibration shown as a C=O bond at 1634.38 cm^−1^ is similar to the uronic acid vibration^18^. The stretching vibration at 1384.62 cm^−1^ indicates CH_3_ bond^19^. The β-furanoside bond stretching vibration at 1154.45 cm^−1^ and 862.045 cm^−121^.

**Table 1:** FVP-C chemical composition results.

Total sugar (%)	Polysaccharides (%)	Reducing sugar (%)	Protein (%)	Uronic acid (%)	Sulfate (%)
41.57 ± 2.99	41.55 ± 2.99	0.02 ± 0.01	0.05 ± 0.01	0.21 ± 0.01	0.98 ± 0.14

### Effect of FVP-S1 and FVP-C on the proliferation and migration of A549 cells

**Figure 2:**
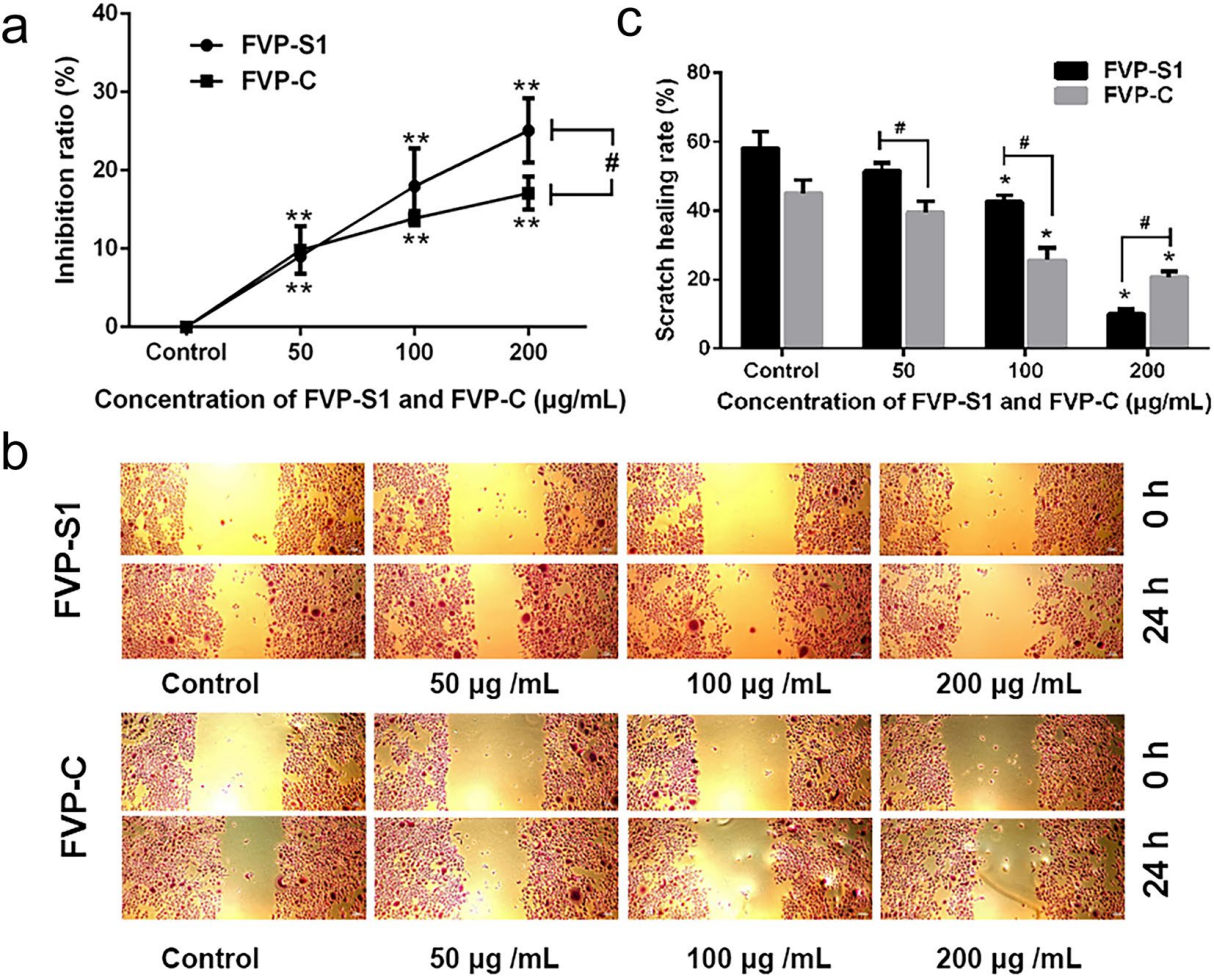
Effect of polysaccharides on proliferation and migration of A549 cells. (**a**) Treatment of A549 cells with different concentrations of FVP-S1 and FVP-C was able to significantly inhibit the proliferation ability of A549 cells, and the best inhibition effect was observed at 200 μg/mL of FVP-S1. (**b,c**) Different concentrations of FVP-S1 and FVP-C were able to inhibit the migratory ability of A549 cells, and the inhibitory effect of FVP-S1 was better than that of FVP-C. Data are shown as mean ± SD. Compared with the control group, an asterisk denotes significance (*p* < 0.05), and two asterisks denote significance (*p* < 0.01). FVP-S1 compared with FVP-C group, #(*p* < 0.05), ##(*p* < 0.01).

To investigate the biological activities of FVP-C and FVP-S1, we treated A549 cells with 50 μg/mL, 100 μg/mL, and 200 μg/mL of FVP-C and FVP-S1, respectively, and detected the proliferation of A549 cells by MTT. The results showed that both FVP-S1 and FVP-C could inhibit cell proliferation in a dose-dependent manner for a certain period of time. The two polysaccharides had the greatest inhibitory effect at 200 μg/mL, with inhibition rates of 25.07% and 17.08%, respectively (Fig. 2a). We next examined the effect of polysaccharides on A549 cell migration and found that in the FVP-S1 treatment group, the healing rate of the untreated cells group was 59.57%, and the treatment rate decreased to 9.38% at 200 μg/mL. Similarly, in the FVP-C treatment group, the healing rate of the control was 40.99%, and the healing rate of the treatment group at 200 μg/mL was the lowest (20.51%) (Fig. 2b,c). The above results indicated that FVP-S1 and FVP-C could indeed inhibit the proliferation and migration of A549 cells. This suggests that FVP-S1 and FVP-C have antitumor effects in vitro, while FVP-S1 may play an important function as a major component of FVP-C.

### Effects of FVP-S1 and FVP-C on the morphology of A549 cells

**Figure 3:**
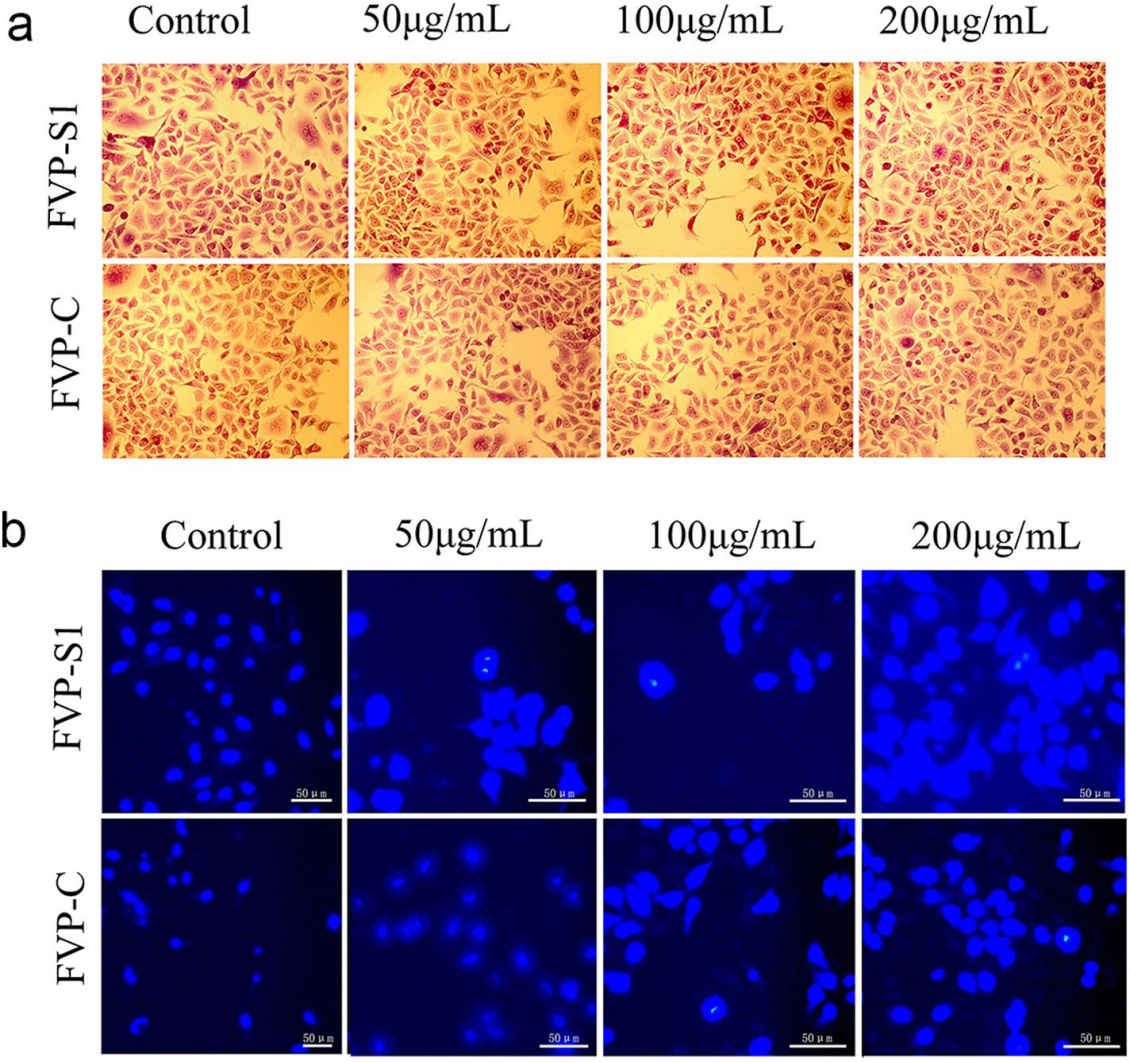
Effects of polysaccharides on A549 cell morphology. (**a**) FVP-S1 and FVP-C can affect the normal morphology of tumor cells. (**b**) Chromosome sheteropythosis phenomenon in cells treated with different concentrations of FVP-S1 and FVP-C.

Subsequently, we investigated the effects of polysaccharides on the morphology of A549 cells, which were observed by crystal violet staining using FVP-S1 and FVP-C treated A549 cells, respectively. Normal-growing A549 cells showed polygonal paving-stone-like shape, whereas A549 cells treated with different concentrations of FVP-S1 and FVP-C showed blunted edges, loose cell arrangement and intercellular junctions, and long spindle-shaped adherence to the wall, which indicated that FVP-S1 and FVP-C affected the normal state of tumor cells (Fig. 3a). In addition, we observed the nuclear morphology of A549 cells by Hoechst33258 staining, and found that chromosomal heteropythosis occurred in cells treated with different concentrations of FVP-S1 and FVP-C, and the heteropythosis phenomenon was more pronounced in the FVP-S1-treated group. The heteropythosis phenomenon was more obvious in the FVP-S1 treated group (Fig. 3b).

### FVP-S1 and FVP-C induced apoptosis in A549 cells

**Figure 4:**
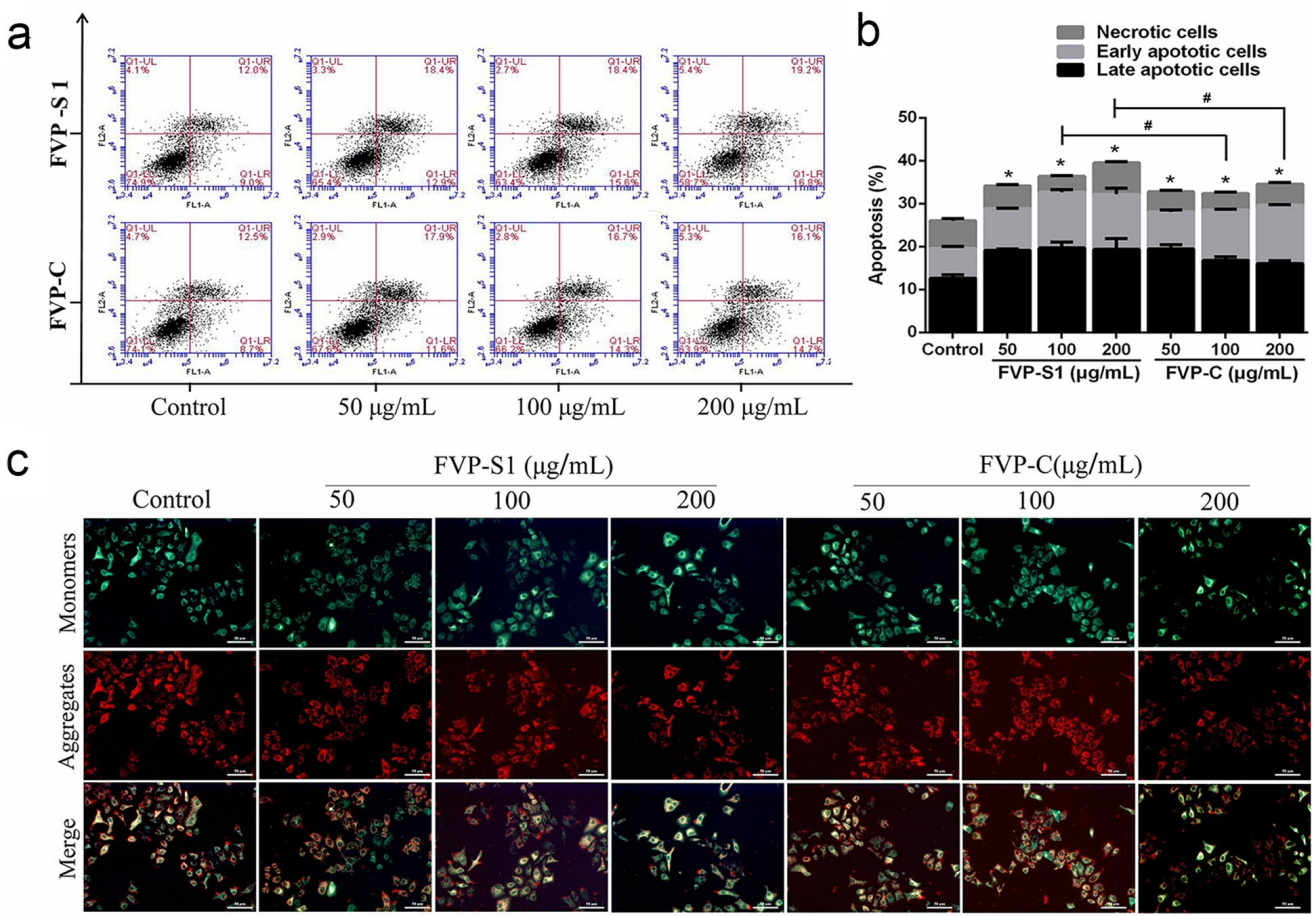
FVP-S1 and FVP-C respectively induced apoptosis in A549 cells. (**a**)Treated cells were stained with Annexin V-FITC/PI, and detected by flow cytometry. (**b**) Column bar graph of apoptotic cells. Surviving cells (Annexin V−/PI−, lower left); early apoptotic cells (Annexin V+/PI−, lower right); Late apoptotic cells (Annexin V+/PI+, upper right); Necrotic cells (Annexin V+/PI−, upper left). The experiments were repeated three times (n = 3). ‘*’ indicates a significantly difference from the control group at the *P* < 0.05 levels. FVP-S1 compared with FVP-C group, #(*p* < 0.05), ##(*p* < *0.01*). (**c**) FVP-S1 and FVP-C induced collapse of mitochondrial membrane potential (MMP) in A549 cells.

MTT and cell migration data showed that FVP-S1 and FVP-C could effectively inhibit the progression of A549 cells, thus we further investigated whether FVP-S1 and FVP-C inhibited the proliferation of A549 cells by apoptosis, flow cytometry was used for A549 cell apoptosis ratio was detected (Fig. 4a). Compared with the control group, the treatment of FVP-S1 and FVP-C at different concentrations (50, 100, and 200 μg/mL) increased the number of early and late apoptotic cells, and the total number of apoptotic cells increased significantly. The apoptotic rate of the control group was 26.03%, and the highest apoptotic rate of the FVP-S1 treatment group at 200 μg/mL was 39.50% (*P* < 0.05). The highest apoptosis rate in the 200 μg/mL FVP-C treatment group was 34.57% (*P* < 0.05, Fig. 4b). The above results confirmed that FVP-S1 and FVP-C had induced apoptosis of A549 cells and that the ratio of FVP-S1 to promote apoptosis of A549 cells is greater than that of FVP-C at polysaccharide concentrations of 100 and 200 μg/mL.

### Effect of FVP-S1 and FVP-C on the mitochondrial membrane potential (MMP) of A549 cells

In the present study, the change of mitochondrial membrane potential was observed under a fluorescent inverted microscope using a fluorescent probe (JC-1) to confirm whether FVP-S1 and FVP-C induce apoptosis through the mitochondrial pathway. Compared with the control group, the red fluorescence level of cells treated with FVP-S1 and FVP-C were significantly decreased, while the green fluorescence was significantly increased (Fig. 4c). The above data indicate that FVP-S1 and FVP-C could indeed induce mitochondrial membrane potential collapse.

### Effects of FVP-S1 and FVP-C on the expression of mRNA related to apoptosis.

**Figure 5:**
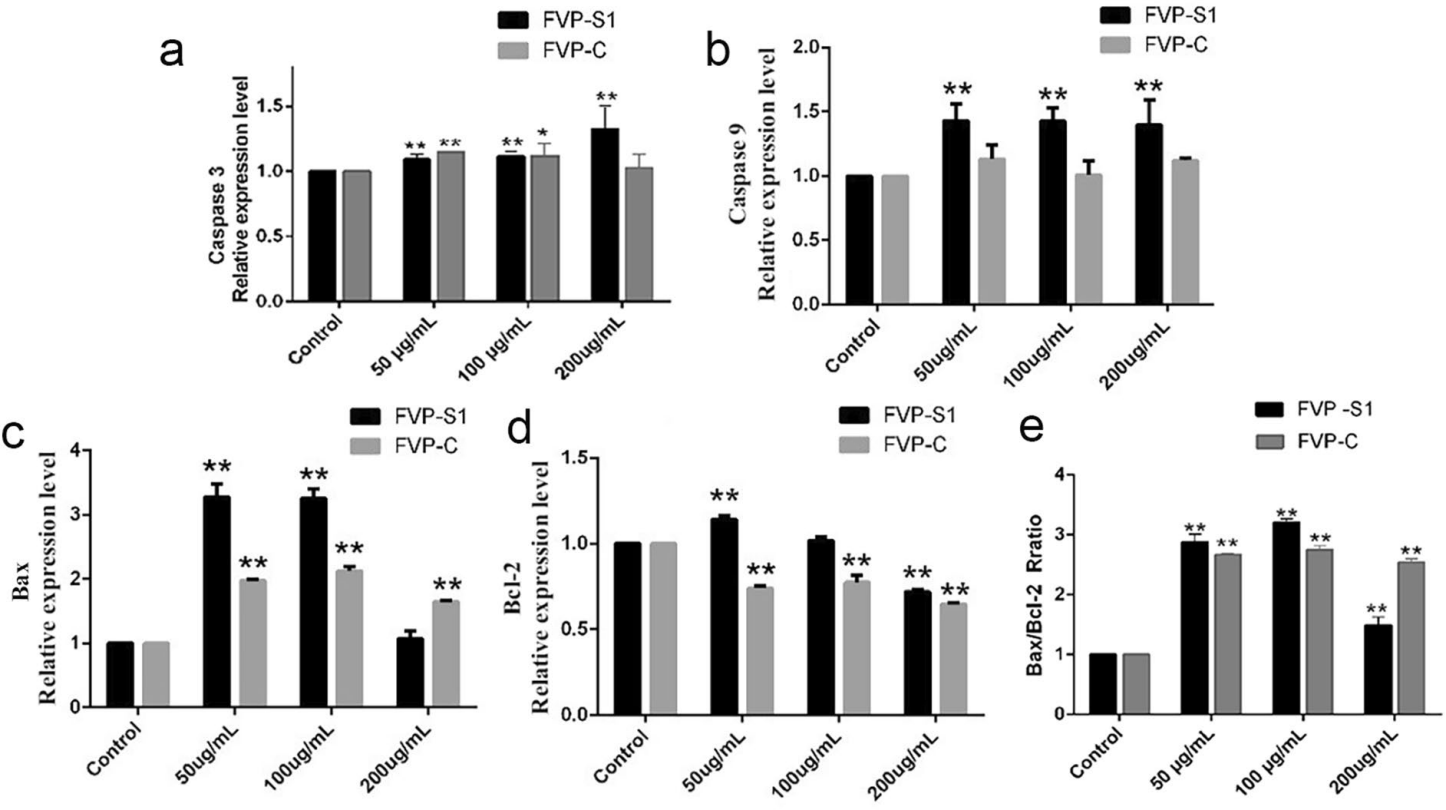
Effects of FVP-S1 and FVP-C on the expression levels of apoptosis-related genes. (**a**) The FVP-S1 and FVP-C treatment groups significantly increased the expression level of caspase 3. (**b**) The FVP-S1 and FVP-C treatment groups significantly increased the expression level of caspase 9. (**c**) The FVP-S1 and FVP-C treatment groups significantly increased the expression level of Bax. (**d**) The FVP-S1 and FVP-C treatment groups significantly increased the expression level of Bcl-2. (**e**) The FVP-S1 and FVP-C treatment groups significantly increased the ratio of Bax to Bcl-2.

To investigate the effects of FVP-S1 and FVP-C on apoptosis-related genes in A549 cells, we detected the mRNA expression of Caspase-9, Caspase-3, Bax, and Bcl-2-related genes in A549 cells treated with FVP-S1 and FVP-C by RT-qPCR. The results of RT-qPCR showed that the expression of caspase-3 and caspase-9 mRNA in A549 cells treated with FVP-S1 was up-regulated, and the relative expression in the treated group at 200 μg/mL was the highest (Fig. 5a,b). Afterwards, the detection of the Bax and Bcl-2 mRNA revealed that both FVP-C and FVP-S1 increased the expression of Bax gene and decreased the expression level of the Bcl-2 gene (Fig. 5c,d), which significantly increased the ratio of Bax to Bcl-2(Fig. 5e).

### Effects of FVP-S1 and FVP-C on the expression of proteins related to apoptosis

**Figure 6:**
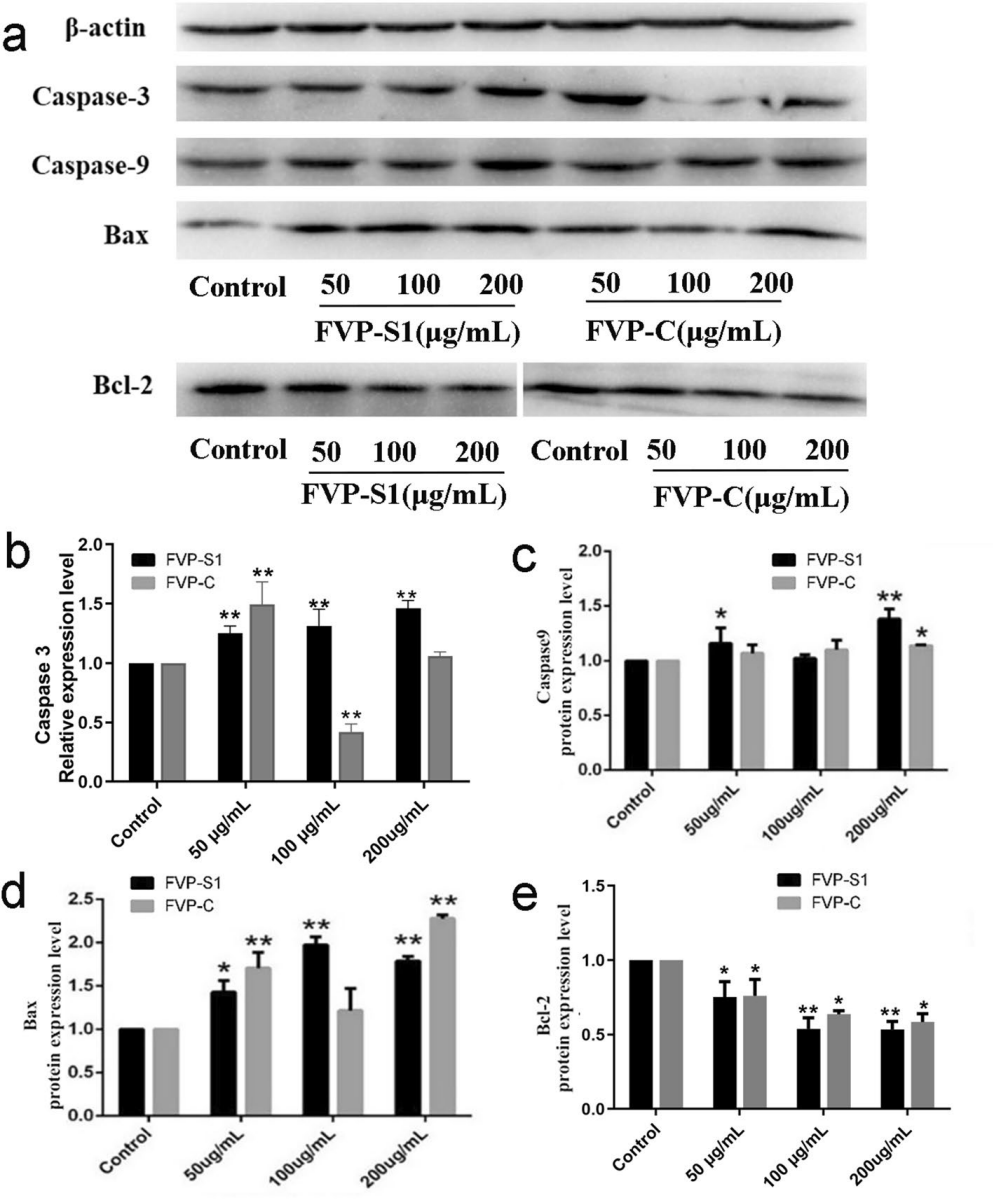
Effects of FVP-S1 and FVP-C on the levels of apoptosis-related proteins. (**a**) Effect of FVP-S1 and FVP-C on the protein expression of Caspase-3, Caspase-9, Bax and Bcl-2 in A549 cells. Original blots are presented in Supplementary Fig. 1–6 (**b**–**e**) A gray value analysis of Caspase-3, Caspase-9, Bax, and Bcl-2 proteins. All of the above protein images are based on the molecular weight of different proteins after cutting the gel and transferring it to PVDF membrane for antibody incubation, and finally for color development and observation. Data are shown as mean ± SD. An asterisk denotes significance (*p* < 0.05), two asterisks denote significance (*p* < 0.01).

Western blot results showed that the expression of Caspase-9, Caspase-3, and Bax, protein in A549 cells treated with FVP-S1 and FVP-C increased in a dose-dependent manner (Fig. 6a). The results show that FVP-S1 and FVP-C treatment groups significantly Caspase-3 and Caspase-9 protein expression levels (Fig. 6b,c). When apoptosis occurs in a cell, firstly Caspase-9 is activated, and the activated Caspase-9 will further activate the execution protein Caspase-3, thus causing cell apoptosis. Therefore, our results show that FVP-S1 and FVP-C did have an effect of promoting apoptosis in A549 cells.

The Western Blot results of Bax and Bcl-2 were consistent with the RT-qPCR results, and both FVP-C and FVP-S1 increased the expression of the Bax gene and decreased the expression level of the Bcl-2 gene (Fig. 6d,e), which significantly increased the ratio of Bax to Bcl-2. In summary, we found that these results were consistent with the mitochondrial results, which further verified that FVP-S1 and FVP-C could regulate apoptosis in A549 cells through the mitochondrial apoptotic pathway.

## Discussion

FVP is a natural active ingredient in *Flammulina velutipes* and has potential applications in the food and pharmaceutical industries. There was evidence to show that FVP has immunomodulatory, antibacterial, and antioxidant activities. In this study, we extracted the water-soluble polysaccharide FVP-C from *Flammulina velutipes*. Previous studies in our laboratory have shown that FVP-C consists of mannose, gluconic acid, glucose, galactose, galactose, xylose, and fructose. In addition, FVP-C is a crude polysaccharide of *Flammulina velutipes* with a heterogeneous composition, and its molecular weight size is expressed by the average molecular weight. The average molecular weight of FVP-C is 1972 kDa, of which the polysaccharide of 1045–1230 kDa contains about 0.5%, the polysaccharide of 1230–2150 kDa contains about 45.2%, the polysaccharide of 2150–2260 kDa contains about 48.4%, and the polysaccharide of 1230–2150 kDa contains about 1.5%. polysaccharide contains about 48.4%, and 2260–238.672 kDa contains about 5.9%^16^. Further analysis revealed that FVP-S1 accounted for the largest proportion of FVP-C. By structural analysis, we found that FVP-C and FVP-S1 belonged to acidic polysaccharides. There was evidence showed that water-soluble polysaccharide and acidic polysaccharides can promote tumor cell apoptosis^20,21^. A galacturonic acid-containing flower bud polysaccharide TFPB1, which can promote the apoptosis-related genes and protein expression levels of Caspase-3, Bax, and reduced Bcl-2 expression in cells, which indicated that TFPB1 has antitumor effects^15^. Therefore, in order to investigate the antitumor activity of FVP-C and to clarify whether FVP-S1 is an effective active ingredient, we used FVP-C and FVP-S1 to treat lung cancer A549 cells respectively. The results showed that both groups were able to inhibit the proliferation and migration of A549 cells, and at the same time change the cell morphology and make the nucleus crumple, which indicated that the polysaccharide had antitumor activity, at the same time, the antitumor effect of FVP-S1 was better than that of FVP-C, and the inhibition effect was more obvious.

Apoptosis is considered to be an important mechanism to prevent the emergence of tumors, and one of the characteristics of tumor cells is the inhibition of apoptosis^22,23^. Therefore, in order to investigate the effects of FVP-C and FVP-S1 on the apoptosis of A549 cells, we detected the apoptosis of A549 cells by flow cytometry, and found that different concentrations of FVP-C and FVP-S1 could cause apoptosis of A549 cells, among which the effect of FVP-S1 was more obvious than that of FVP-C, and the apoptosis rate was highest in the 200 μg/mL FVP-S1 treatment group, which indicated that polysaccharides could promote apoptosis in A549 cells, in which FVP-S1 played an important role.

The decrease in the number of apoptotic cells is closely related to the occurrence of cancer, autoimmune diseases, and neurodegenerative diseases^24^. In response to this phenomenon, induction of apoptosis has become the main strategy for the treatment of such diseases^22^. Mitochondrial membrane potential usually decreases during apoptosis^25^, which could lead to changes in mitochondrial permeability. Therefore, we examined the changes of FVP-C and FVP-S1 on the mitochondrial membrane potential of A549 and found that FVP-S1 and FVP-C indeed induced mitochondrial membrane potential collapse.

To further confirm this opinion, we investigated the changes in mRNA and protein expression of apoptosis-related genes Caspase-9, Caspase-3, Bax, and Bcl-2. Caspase protein is an important molecular marker of cell apoptosis. Caspase-9 acts as a promoter in response to upstream apoptotic signals in the mitochondrial apoptotic pathway. Once the promoter Caspase-9 receives an apoptotic signal, it activates the Caspase-3, which is downstream of it, while Caspase-3 is a key protein that performs apoptosis and plays an important role in performing apoptosis tasks^26^. Meanwhile, as a star factor that regulates mitochondrial apoptosis, the Bcl-2 family plays an important role in the apoptosis. It can adjust the mitochondrial membrane potential^27^, thereby releasing cytochrome C from the mitochondrial membrane to the cytosol and binding to apoptotic protease activating factor 1 (Apaf-1). Immediately, recruits Caspase-9 in the cytoplasm and then activates Caspase-3 to perform apoptosis^28^. According to their different functions, the Bcl-2 family can be divided into two categories: pro-apoptotic factors (such as Bax) and anti-apoptotic factors (such as Bcl-2)^29^. The expression levels of Bax and Bcl-2 in the cells showed opposite trends. The increase of Bax content during cell apoptosis promoted the release of cytochrome C^30^. At this time, the content of Bcl-2 in the cell showed a downward trend, and its ability to inhibit the release of cytochrome C combined with the outer membrane of mitochondria was weakened. Therefore, increasing Bax content in cells and decreasing Bcl-2 content can play a role in promoting apoptosis.

Through the detection of these genes, we found that FVP-S1 and FVP-C were able to increase the expression levels of caspase-3 and caspase-9 mRNA and proteins, and increase the genes and proteins expression of the proapoptotic factor Bax. It also reduced the expression of anti-apoptotic factor Bcl-2, as well as increased the ratio of Bax and Bcl-2, mitochondria, and also increased the mitochondrial membrane potential of A549 cells and promoted apoptosis.

## Conclusion

In conclusion, we found that acidic *Flammulina velutipes* polysaccharide FVP-S1 and *Flammulina velutipes* crude polysaccharide FVP-C were able to induce apoptosis in A549 cells through the mitochondrial pathway, in which FVP-S1 is the main active component in FVP-C, which plays an important role in inhibiting tumor cells.

## Materials and methods

### Materials and chemicals

The Fresh fruiting bodies of *Flammulina velutipes* were collected at Changchun Gaorong Biological Technology Co., Ltd. A549 cells were presented by the College of Life Sciences of Jilin University. DEAE-Sepharose Fast Flow and Sephadex G-100 were purchased from GE Healthcare Life Science (Fairfield City, Connecticut, USA). Hoechst 33258 diet, JC-1 mitochondrial membrane potential detection assay kit was obtained from Beyotime Biotechnology (Shanghai, China). Annexin V-FITC/PI apoptosis detection kit was acquired from BD company (Becton Dickinson, Bedford, MA, USA), antibodies against Caspase-3, Caspase-9, Bax, Bcl-2 and β-actin were obtained from Cell Signaling Technology (Beverly, MA, USA).

### Isolation and purification of polysaccharide from Flammulina velutipes

After air drying, the *Flammulina velutipes* fruiting body were ground and passed through a 100-meshes sieve for use. 95% ethanol was immersed in the fruiting body at 4 °C overnight to remove lipids and pigments, and the *Flammulina velutipes* fruiting body were extracted twice by distilled water at 100 °C for 3 h. The liquid-to-material ratio was 41:1. The combined two aqueous extracts were concentrated at 60 °C, slowly added with 95% ethanol to a final concentration of 75%, and precipitated overnight at 4 °C. The precipitate was collected and dissolved in distilled water, and then the protein was removed by the Sevag method. The samples were dialyzed for 48 h, and the crude polysaccharides were lyophilized and designated as FVP-C. FVP-C was separated by DEAE-Sepharose Fast Flow ion exchange chromatography column (3.6 cm × 20 cm), and 0, 0.1, 0.3 M NaCl were used as the elution phase. The eluate was collected and dialyzed for 48 h. The liquids were named FVP-W, FVP-S, and FVP-HS, respectively. FVP-S on Sephadex G-100 molecular sieves (2.6 cm × 100 cm) was eluted using 0.1 M NaCl. The eluate containing FVP-S was obtained and dialyzed for 48 h. The dried polysaccharide powder obtained was named FVP-S1.

### Physicochemical and structural analysis of polysaccharide

#### Chemical component analysis

Chemical method to detect the content of substances in FVP-C. The total carbohydrate content was determined by the phenol–sulfuric acid method based on glucose. The reducing sugar content was determined by the 3,5-Dinitrosalicylic acid (DNS) method using glucose as a standard. The protein content was determined using the Coomassie Brilliant Blue G-250 method based on bovine serum albumin. Uronic acid content was determined by the method of m-hydroxybiphenyl using galacturonic acid as a standard. The content of sulfate was determined by the ruthenium chloride-gelatin turbidimetric method using potassium sulfate as the standard.

#### Molecular weight analysis

The molecular weight of FVP-S1 was determined by high performance gel permeation chromatography (HPGPC). The HPGPC is operated by the HPGPC system, which was equipped with the TSK-Gel G3000PWXL Column (300 × 7.8 mm), Shimadzu LC-10 ATVP pump, Shimadzu RID-10A differential detector. The injection sample volume was 20 μL the that concentration was 5 mg/mL. HPGPC system using 0.2 mol/L NaCl as the mobile phase at a flow rate of 0.6 mL/min.

#### Monosaccharide composition analysis

Weigh 5 mg of FVP-S1 in a hydrolysis bottle, add 1 M hydrochloric acid anhydrous methanol solution, hydrolyze at 80 °C for 16 h, then add 2 M trifluoroacetic acid (TFA, C_2_HF_3_O_2_), hydrolyze at 120 °C for 1 h, transfer the hydrolysate to the evaporating dish and add the appropriate amount. Water ethanol was evaporated to dryness in a water bath to remove TFA. The hydrolyzed FVP-S1 was subjected to high performance liquid chromatography (HPLC) analysis. The HPLC system was equipped with Amethyst C18-H (4.6 × 250 mm), Shimadzu LC-20AT pump, and Shimadzu evaporative light detector (detection wavelength 245 nm). Water and acetonitrile (80:20 (v/v)) were used as the mobile phase at a flow rate of 0.85 mL/min.

#### Infrared spectrum analysis

Infrared spectra were analyzed for FVP-S1 and FVP-C, respectively. FVP-S1 and FVP-C were compressed by Potassium bromide (KBr), compressed into tablets, and then scanned with the HIMADZU-8400 s Fourier transform infrared spectrometer for 32 times in the range of 4000–500 cm^−1^.

### Cell culture

A549 cells were obtained from Jilin University (Jilin, China). The cells were cultured in Dulbecco’s Modified Eagle Medium (DMEM) containing with 10% fetal bovine serum (FBS) and cultured in an incubator at 37 °C, 5% CO_2_ and 95% humidity.

### Inhibition of cell proliferation assay

Cell proliferation inhibition was detected by MTT assay. The cells (10^4^ cells/well) were seeded in a 96-well plate, and cells were treated with 150 μL of complete medium respectively containing FVP-S1 and FVP-C (final concentrations of 0, 50, 100, 200 μg/mL) for 24 h. Then add 15 μL of MTT (5 mg/mL) for 4 h at 37 °C, aspirate the supernatant, add 150 μL of DMSO (dimethyl sulfoxide) for 30 min, and measure the absorbance at 490 nm. The tumor cell inhibition rate was as follows: inhibition rate = (1-A/B)* 100%, where A is the absorbance of polysaccharide of *Flammulina velutipes*, and B is the absorbance of the control group.

### Cell scratch

Cell scratch assays detect cell migration. First, we drew 3 uniform horizontal lines on the back of six-well plates. Then, inoculated the cells (5 × 10^5^ cells/well) in a six-well plate, and incubated overnight. On the second day, we used the tip of the pipette to draw vertical lines on the cultured cells. Later, the scraped cells were washed by using phosphate buffer saline (PBS). Afterwards, add 2.5 mL of complete medium respectively containing FVP-S1 and FVP-C (final concentration of 0, 50, 100, 200 μg/mL) for 24 h. Pre-cooled 70% ice ethanol was fixed at 4 °C for 10 min, then wash the cells twice with PBS, added 400 μL of crystal violet (1% mass fraction) for 10 min and washed once with PBS, optically inverted^31–33^. Take a photo with a microscope. The scratch healing rate was as follows: scratch healing rate = (B-A)/B* 100%, where A is the 0 h line at the unhealed area, and B is the 0 h line area.

### Cell morphology detection

A549 cells were inoculated in six-well plates at a density of 5 × 10^5^, and when the cells were in the logarithmic growth phase, 2 mL of complete medium (0, 50, 100, 200 μg/mL) containing different concentrations of FVP-S1 and FVP-C were added to treat the cells for 24 h, and the medium was removed and washed twice with PBS, and the cells were fixed by adding 2 mL of pre-cooled 70% iced ethanol at 4 °C for 10 min in each well, then washed twice with PBS. After the cells were washed twice with PBS, 400 μL of crystal violet staining solution (1% by mass) was added and the cells were stained for 10 min and washed once with PBS, and then observed and photographed with an ordinary light microscope (Axiocam 506 color).

### Hoechst 33258 staining

A549 cells were inoculated in six-well plates at a density of 5 × 10^5^, and 2 mL of complete medium (0, 50, 100, 200 μg/mL) containing different concentrations of FVP-S1 and FVP-C were added to treat the cells when the cell growth was in the logarithmic growth phase. First, the adherent cells were washed twice with pre-cooled PBS, and 1 mL of 4% formaldehyde solution was added to fix the cells at 4 °C for 10 min, then the cells were washed twice with pre-cooled PBS, and the cells were stained by adding 100 μL of Hoechst 33258 working solution after removing as much as possible from the PBS for 10 min at room temperature, and then photographed for observation by fluorescence inverted microscope (Axiocam 506 color). Fluorescence inversion microscope (Axiocam 506 color) was used to observe and take photos.

### Analysis of apoptosis by flow cytometry

Apoptosis was detected using the Annexin V-FITC/PI Apoptosis Kit (BD, USA). The cells (5 × 10^5^ cells/well) were seeded in a Petri dish (6 cm), add 5 mL of complete medium respectively containing FVP-S1 and FVP-C (final concentration of 0, 50, 100, 200 μg/mL) for 24 h. The cells were harvested and treated according to the apoptotic kit instructions, incubated for 20 min at room temperature in the dark, and then analyzed by flow cytometry using Accuri C6 (BD, USA).

### Detection of the mitochondrial membrane potential (MMP)

Mitochondrial membrane potential (MMP) was measured using a JC-1 kit. Briefly, seed cells (5 × 10^5^ cells/well) in a Petri dish (6 cm), add 5 mL of complete medium respectively containing FVP-S1 and FVP-C (final concentration 0, 50, 100, 200 μg/mL) for 24 h. The cells were collected, added with JC-1 staining solution and incubated at 37 °C for 20 min in the dark, then the cells were washed twice with JC-1 working solution, and photographed by fluorescence inverted microscope (Axiocam 506 color).

### RT-qPCR

The relative expression level of mRNA in cells was detected by RT-qPCR. The cells were seeded in a Petri dish (10 cm), and cells were treated with 10 mL of complete medium respectively containing FVP-S1 and FVP-C (final concentrations of 0, 50, 100, 200 μg/mL) for 24 h. Total RNA was extracted by the Trizol method, and Reverse Transcription was performed by PrimeScript RT reagent kit (Takara Biotechnology Co., Ltd.). At the same time, the caspase-3, caspase-9, Bax, Bcl-2 and β-Actin gene sequences reported by GenBank were searched, and the above gene-specific primers were designed using Primer5.0. RT-qPCR reagents, cDNAs and primers were added according to the instructions of SYBR Premix Ex TaqTM II Kit (Takara Biotechnology Co., Ltd.) and tested on an Agilent Mx3000P (Agilent, USA). The relative expression level of the mRNA of the above gene was calculated using the 2^−ΔΔCt^ method. The specific calculation formula is as follows: △△Ct = (Ct target gene − Ct internal reference gene) × − (Ct target gene − Ct internal reference gene) control group. Among them, β-actin is used as an internal reference gene, and x is an arbitrary sample. Primer sequences are shown in Table 2.

**Table 2:** A list of primers used in this study.

Gene	Sense	Antisense
caspase-3	AAGGCAGAGCCATGGACCAC	CTGGCAGCATCATCCACACATAC
caspase-9	GAACTAACAGGCAAGCAGCAAA	CGACATCACCAAATCCTCCA
Bcl-2	TGGACAACCATGACCTTGGAC	GTGCTCAGCTTGGTATGCAGAA
Bax	GGATGCGTCCACCAAGAAG	GCAAAGTAGAAAAGGGCGACA
β-actin	TGGCACCCAGCACAATGAA	CTAAGTCATAGTCCGCCTAGAAGCA

### Western blotting assay

Western Blot was used to detect the effects of FVP-S1 and FVP-C on apoptosis-related proteins (Caspase-3, Caspase-9, Bax, Bcl-2) in A549 cells. Inoculate A549 cells in a Petri dish (10 cm). After the cells were attached, add 10 mL of complete medium respectively containing FVP-S1 and FVP-C (final concentrations of 0, 50, 100, 200 μg/mL). After 24 h of cell extraction, total protein was extracted and 12% or 15% SDS-PAGE was used to separate apoptosis-related proteins. Based on the molecular weights of the different proteins, the gels were trimmed according to the specific positions shown by the Marker, after which it was transferred to PVDF membranes for incubation with antibodies to the corresponding proteins, chemiluminescent positive bands on the membrane were detected using ChemDoc MP (Bio-Rad). The Quantity One.462 software analyzes the gray value of the positive band and calculates the relative expression level, using the relative expression of β-actin protein as an internal standard control group. Antibody Information: Antibodies for this experiment were purchased from Cell Signaling Technology, Inc (Danvers, MA, USA), Caspase-3 Antibody (CST#9662), Caspase-9 Antibody (CST#9502), Bax Antibody (CST#2774), Bcl-2 Antibody (CST#2872). The relative expression of protein uses the following formula: relative expression of protein = A/B, where A is the gray value of the target protein, and B is the gray value of the internal reference protein.

### Statistical analysis

Statistical analysis was performed using SPSS Statistics 22, and the data were reflected in the article as mean ± SD (standard deviation). The One-way analysis of variance (ANOVA) with Duncan and the Least Significant Difference test (LSD) was used to determine the component's display. Sexuality, and *P* < 0.05 as a statistical significance test. Histograms and scatter plots were drawn using GraphPad Prism.6.

### Supplementary Information

Supplementary Figures.

## Data Availability

The original contributions presented in the study are included in the article, and further inquiries can be directed to the corresponding author.
