# Supplementary material for: Effect of Flammulina velutipes polysaccharide on mitochondrial apoptosis in lung adenocarcinoma A549 cells
Source: Sci Rep. 2024 Jul 12;14:16102. doi: 10.1038/s41598-024-57211-x (PMC11245558; doi:10.1038/s41598-024-57211-x)
Supplement: Supplementary file 1 — Supplementary Figures. [file 41598_2024_57211_MOESM1_ESM.pdf]

Supplemental Information

Effect of Flammulina velutipes polysaccharide on mitochondrial apoptosis in lung adenocarcinoma A549 cells

Fei Zhao [1], Dan-yang Chen [1,2], Bo Jing [1], Yu Jiang [1], Lan-yue Liu [1] and Hui Song [1, 3,*]

The original Western blotting images of Fig. 6a in the text is as follows:

β-actin

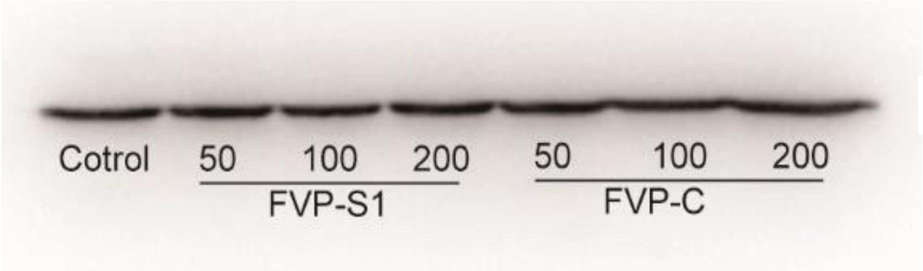

45 kDa

Supplementary Fig. 1 Protein expression levels of β-actin after FVP-C and FVP-S1 treatment.

Caspase-3

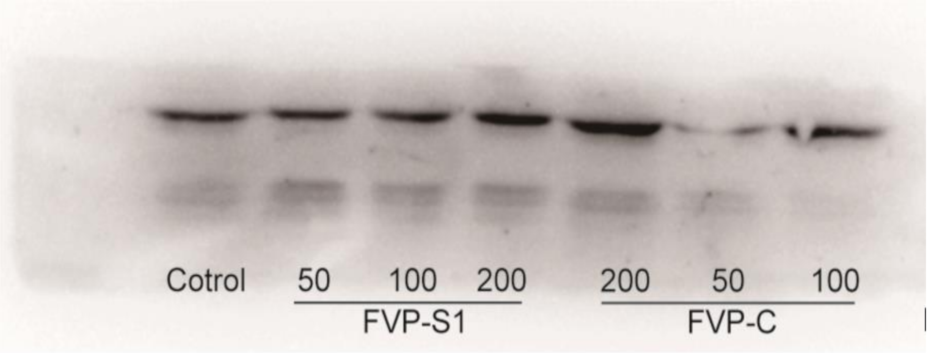

35 kDa

Supplementary Fig. 2 Protein expression levels of Caspase-3 after FVP-C and FVP-S1 treatment.

Caspase-9

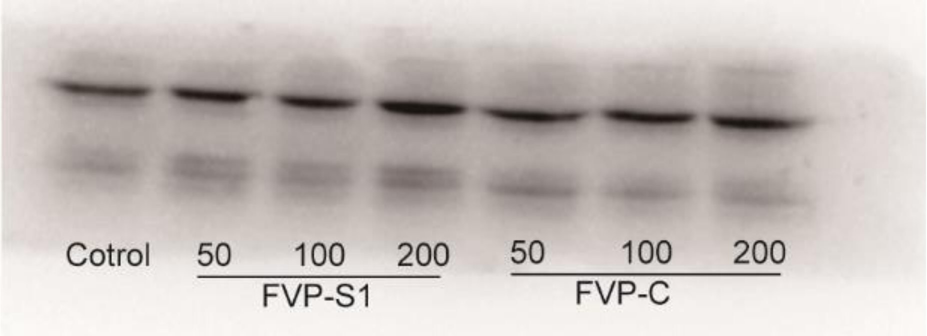

47 kDa

Supplementary Fig. 3 Protein expression levels of Caspase-9 after FVP-C and FVP-S1 treatment.

Bax

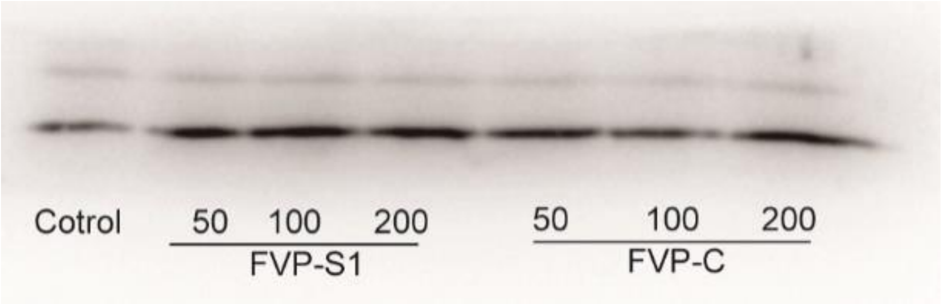

20 kDa

Supplementary Fig. 4 Protein expression levels of Bax after FVP-C and FVP-S1 treatment.

Bcl-2

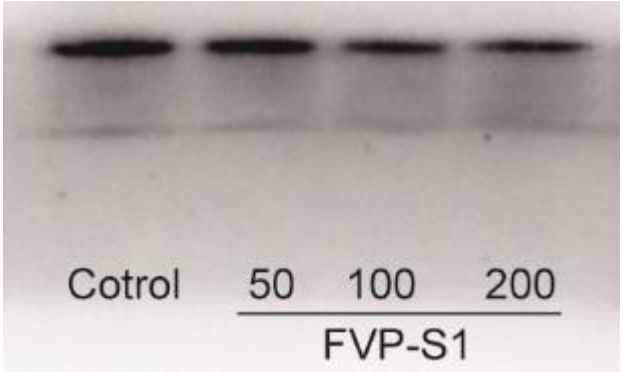

28 kDa

Supplementary Fig. 5 Protein expression levels of Bcl-2 after FVP-S1 treatment.

Bcl-2

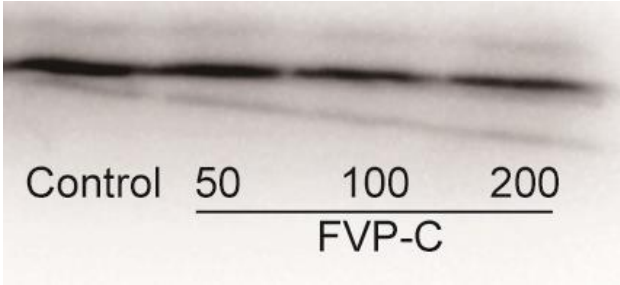

28 kDa

Supplementary Fig. 6 Protein expression levels of Bcl-2 after FVP-C treatment.
